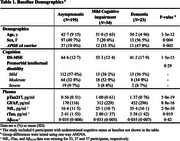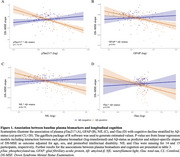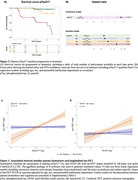# Prediction of future tau accumulation and cognitive decline in Down Syndrome using plasma biomarkers

**DOI:** 10.1002/alz70856_099360

**Published:** 2025-12-24

**Authors:** Shorena Janelidze, Lyduine E. Collij, Niklas Mattsson‐Carlgren, Alex Antill, Charles M Laymon, Ira T. Lott, H. Diana Rosas, Davneet S Minhas, Weiquan Luo, Shahid Zaman, Mark Mapstone, Florence Lai, Sigan L Hartley, Beau Ances, Rik Ossenkoppele, Bradley T Christian, Benjamin L Handen, Oskar Hansson

**Affiliations:** ^1^ Clinical Memory Research Unit, Lund University, Lund, NA, Sweden; ^2^ Clinical Memory Research Unit, Department of Clinical Sciences Malmö, Faculty of Medicine, Lund University, Lund, Sweden; ^3^ Clinical Memory Research Unit, Department of Clinical Sciences Malmö, Faculty of Medicine, Lund University, Sweden, Lund, Sweden; ^4^ Memory Clinic, Skåne University Hospital, Malmö, Skåne, Sweden; ^5^ Lund University, Lund, NA, Sweden; ^6^ Department of Radiology and Bioengineering, University of Pittsburgh, Pittsburgh, PA, USA; ^7^ University of California, Irvine, Irvine, CA, USA; ^8^ Massachusetts General Hospital, Charlestown, MA, USA; ^9^ University of Pittsburgh, Pittsburgh, PA, USA; ^10^ University of Cambridge, Cambridge, United Kingdom; ^11^ Harvard/Massachusetts General Hospital, Boston, MA, USA; ^12^ University of Wisconsin‐Madison, Madison, WI, USA; ^13^ Washington University in St. Louis School of Medicine, St. Louis, MO, USA; ^14^ Alzheimer Center Amsterdam, Neurology, Vrije Universiteit Amsterdam, Amsterdam UMC location VUmc, Amsterdam, Netherlands; ^15^ Waisman Center, University of Wisconsin‐Madison, Madison, WI, USA

## Abstract

**Background:**

Plasma biomarkers of Alzheimer's disease (AD) could improve the prognostic evaluation of individuals with Down syndrome (DS) in both clinical practice and trials. In this study, we aimed to identify plasma biomarkers that best predict changes in AD‐related tau pathology and cognitive decline in DS.

**Method:**

The study comprised 258 adults with DS from the longitudinal ABC‐DS study, with plasma phospho‐tau217 (pTau217), glial fibrillary acidic protein (GFAP), amyloid‐β (Aβ)_42/40_, neurofilament light (NfL), and total‐tau (tTau) assessments (Table‐1). Associations of plasma biomarkers with slopes of global cognitive functioning (Down Syndrome Mental Status Examination scores [DS‐MSE]) and tau‐PET ([^18^F]flortaucipir uptake in temporal meta‐region) were examined using linear regression models. Plasma biomarker associated risk of progression to dementia was determined using survival analysis.

**Result:**

Baseline pTau217, GFAP, NfL, and tTau were all individually associated with longitudinal change of DS‐MSE (pTau217: β=‐0·38; GFAP: β=‐0·40; NfL: β=‐0·34; tTau: β=‐0·19; *p* <0·002, Figure 1) and progression to dementia (pTau217: HR=3·95, *p* <0·001; GFAP: HR=3·87, *p* <0·001; NfL: HR=2·05, tTau: HR=1·75; *p* <0·008). However, in a combined model, only pTau217 remained associated with cognitive deterioration (β=‐0·27, *p* <0·001) and progression to dementia (HR=3·47, *p* <0·001, Figure 2). Associations with tau‐PET slopes (*n* = 62) were significant for baseline plasma pTau217 (β=0·29, *p* = 0·02) and GFAP (β=0·42, *p* = 0·002; Figure 3) individually and in the combined model (pTau217: β=0·24, *p* = 0·04; GFAP, β=0·39, *p* = 0·005). Longitudinal change of plasma pTau217 levels correlated with longitudinal change of cognition (β=‐0·43, *p* <0·001) and tau‐PET (β=0·83, *p* <0·001).

**Conclusion:**

Baseline plasma pTau217 predicted subsequent decline in global cognition and progression to dementia, whereas both pTau217 and GFAP were associated with an increase in tau burden. Longitudinal increase in pTau217 levels was associated with accelerated cognitive decline and tau deposition. These findings suggest that blood pTau217, potentially together with GFAP, could support the prognostic workup of AD in individuals with DS in both clinical practice and trials.